# Lethal and sublethal impacts of membrane-fed ivermectin are concentration dependent in *Anopheles coluzzii*

**DOI:** 10.1186/s13071-024-06287-5

**Published:** 2024-05-16

**Authors:** Monique A. M. Shepherd-Gorringe, Marie W. Pettit, Frances M. Hawkes

**Affiliations:** 1 Medway Centre for Pharmaceutical Science, University of Greenwich, Chatham Maritime, Kent, ME4 4TB UK; 2 Natural Resources Institute, University of Greenwich at Medway, Chatham Maritime, Kent, ME4 4TB UK

**Keywords:** Ivermectin, Endectocides, *Anopheles coluzzii*, Vector control, Survival, Fecundity

## Abstract

**Background:**

Ivermectin is a well-tolerated anthelminthic drug with wide clinical and veterinary applications. It also has lethal and sublethal effects on mosquitoes. Mass drug administration with ivermectin has therefore been suggested as an innovative vector control tool in efforts to curb emerging insecticide resistance and reduce residual malaria transition. To support assessments of the feasibility and efficacy of current and future formulations of ivermectin for vector control, we sought to establish the relationship between ivermectin concentration and its lethal and sublethal impacts in a primary malaria vector.

**Methods:**

The in vitro effects of ivermectin on daily mortality and fecundity, measured by egg production, were assessed up to 14 days post-blood feed in a laboratory colony of *Anopheles coluzzii.* Mosquitoes were fed ivermectin in blood meals delivered by membrane feeding at one of six concentrations: 0 ng/ml (control), 10 ng/ml, 15 ng/ml, 25 ng/ml, 50 ng/ml, 75 ng/ml, and 100 ng/ml.

**Results:**

Ivermectin had a significant effect on mosquito survival in a concentration-dependent manner. The LC_50_ at 7 days was 19.7 ng/ml. The time to median mortality at ≥ 50 ng/ml was ≤ 4 days, compared to 9.6 days for control, and 6.3–7.6 days for ivermectin concentrations between 10 and 25 ng/ml. Fecundity was also affected; no oviposition was observed in surviving females from the two highest concentration treatment groups. While females exposed to 10 to 50 ng/ml of ivermectin did oviposit, significantly fewer did so in the 50 ng/ml treatment group compared to the control, and they also produced significantly fewer eggs.

**Conclusions:**

Our results showed ivermectin reduced mosquito survival in a concentration-dependent manner and at ≥ 50 ng/ml significantly reduced fecundity in *An. coluzzii*. Results indicate that levels of ivermectin found in human blood following ingestion of a single 150–200 μg/kg dose would be sufficient to achieve 50% mortality across 7 days; however, fecundity in survivors is unlikely to be affected. At higher doses, a substantial impact on both survival and fecundity is likely. Treating human populations with ivermectin could be used as a supplementary malaria vector control method to kill mosquito populations and supress their reproduction; however strategies to safely maintain mosquitocidal blood levels of ivermectin against all *Anopheles* species require development.

**Graphical Abstract:**

**Figure:**
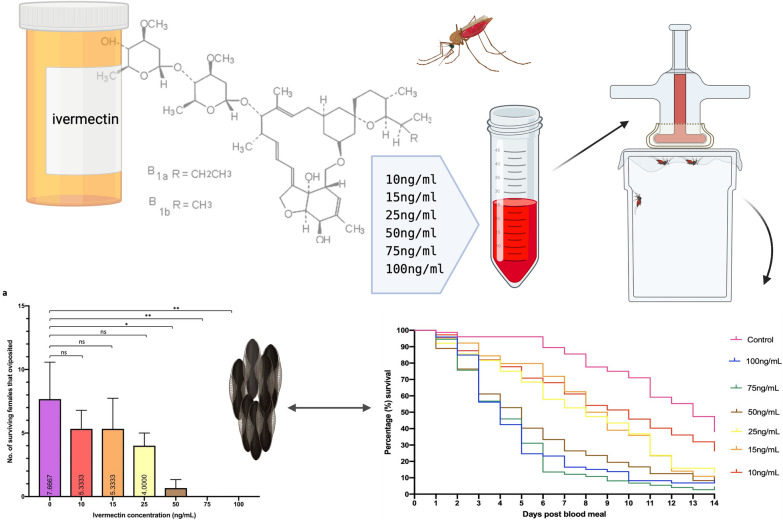

## Background

Ivermectin is an historically well tolerated drug licenced for human use under the brand Stromectol^®^ as an anti-parasitic medication and is also available as a generic drug. It has already had a positive impact on human health as the essential mainstay of two global disease elimination programmes against onchocerciasis and lymphatic filariasis, both endoparasitic infections [1, 2]. However, its broad-spectrum endectocide activity means ivermectin is capable of killing both endo- and ectoparasites when administered to the host [1] and has also been shown to cause mortality in various mosquito species when ingested through a blood meal, including across the *Anopheles* genus of malaria vectors [2]. Repurposing ivermectin as a vector control tool to support malaria elimination therefore holds promise for further extending the profile of the drug in its role in human health.

Ivermectin is a macrocyclic lactone derived from avermectins naturally produced by the bacteria *Streptomyces avermitilis* [3] and has a novel mode of action compared to other insecticides currently used in malaria vector control [4]. The broad-spectrum activity of ivermectin in a number of biological systems is due to the primary mode of action as an activator of glutamate-gated chloride channel receptors (GluCIRs) [5], which are found in both nematodes and invertebrates such as mosquitoes. Once bound to these receptors, ivermectin affects the neuro-muscular junction causing death by paralysis [6]. The absence of GluCIRs in mammals may account for the excellent safety profile and relative lack of side effects in human hosts [7].

The use of ivermectin in mass drug administrations (MDA) for the purpose of malaria vector control has been gaining traction in high burden countries in sub-Sharan Africa due to the drug’s ability to kill mosquitoes that have blood-fed from ivermectin treated patients [4]. This is a promising development in efforts to achieve the ambitious goals for malaria control set for 2030 by the World Health Organisation [8]. MDA of ivermectin has the potential to target both indoor and outdoor biting mosquitoes and help curb residual malaria transmission, which is the result of insecticide resistance and adaptive mosquito behaviours that allow vectors to thrive and sustain transmission despite good uptake of conventional vector control methods, such as bed nets or insecticide residual spraying [9]. Several studies have published data on in vitro dose-response or viability assays of mosquito colonies exposed to ivermectin-laced blood meals [10–12]. Some of these studies have also sought to deduce the effect of ivermectin on mosquito fecundity (reproductive output) in addition to survivorship, with a view that ivermectin may still suppress vector population density if host blood ivermectin levels fall below the lethal threshold and/or the killing effect is limited to 14 days post ingestion of the ivermectin blood meal [13].

Lethal and sublethal impacts of ivermectin in mosquitoes have been previously documented both in vitro and in vivo across several studies, having been assessed in the field through ivermectin MDA [14] and under laboratory conditions [10, 11, 15] and together they estimate the mosquitocidal effects of ivermectin for a number of mosquito species. In the field, a randomised, double-blind placebo-controlled study in Kenya in 2018 by Smit et al. (IVERMAL) [14] found that, compared to control, one ivermectin dose of 300 μg/kg per day for 3 days administered to human participants orally was sufficient to have a 14-day post-feeding mosquitocidal effect on *Anopheles gambiae *sensu stricto that had fed on blood taken from participants 7 days after treatment. The study also found that mosquito mortality remained significantly elevated when mosquitoes were fed blood taken at 28 days post treatment and that as such treatment with 300 μg/kg per day for 3 days showed promise as a potentially suitable dose for vector control. Variance in the in vitro dose-response effect of ivermectin on malaria mosquito colonies in the field versus laboratory reared colonies has been suggested, where wild-type mosquitoes showed greater susceptibility to ivermectin induced mortality compared to laboratory-maintained colony mosquitoes of the same species [12].

Under laboratory conditions ivermectin decreases the daily probability of survivorship in *An. gambiae* species complex up to 14 days post-blood feeding*,* and uptake of sublethal concentrations has been shown to affect mosquito fecundity in *Anopheles* species*,* most recently in *Anopheles arabiensis*. The concentration of ivermectin it takes to kill 50% of mosquitoes (LC_50_) in 7 days seems to vary in *Anopheles* by species, from 3 to 55 ng/ml [25]. A study in *An. gambiae* s.s. found lethal concentrations of membrane-fed ivermectin to be between 6.1 and 15.9 ng/ml (LC_50_ = 15.9 ng/ml, LC_35_ = 12.7 ng/ml, LC_25_ = 10.7 ng/ml, LC_5_ = 6.1 ng/ml) [16]. In *Anopheles darlingi*, lethal concentrations of similarly administered ivermectin were found to be slightly higher, being between 14.8 and 43.2 ng/ml (LC_50_ = 43.2 ng/ml, LC_25_ = 27.8 ng/ml, LC_5_ = 14.8 ng/ml) [10]. A study of the effect of ivermectin concentration on survival over 9 days in *An. arabiensis* found ivermectin to have a significant impact on survival at concentrations between 5 and 80 ng/ml, where survival was found to decrease by 2.3, 3.5, 6.5, 11.5, and 27.9 times that of the control in adult females membrane-fed ivermectin at 5 ng/ml, 10 ng/ml, 20 ng/ml, 40 ng/ml, and 80 ng/ml, respectively [12].

Studies showing the efficacy of ivermectin as a mosquitocide have been broadly focussed on *An. gambiae* s.s., but for ivermectin to be used most effectively as a vector control tool, understanding the effects of ivermectin in all *Anopheles* species is vital to its success. *Anopheles coluzzii* is a primary vector of human malaria in sub-Saharan Africa, but its response to ivermectin has not been established. *Anopheles coluzzii* displays predominantly anthropophilic and endophilic behaviour [17] and is closely related to *An. gambiae* s.s., having only been established as a separate species in 2013 [18]. Despite their genetic similarities, *An. coluzzii* shows greater plasticity in its behaviour and adaptive capacity than its sister species *An. gambiae* s.s.; *An. coluzzii* has been shown to expand its range of peak biting times to avoid insecticide-treated bed nets [19] and shows a greater resistance to DDT and pyrethroid insecticides used for vector control [20–22]. More recently, it has adapted to urban environments, where it has spread into the main cities of Central Africa [17], in part because of an increased tolerance to organic pollution and insecticides. The spread of a major vector species like *An. coluzzii* to urban settings is a major threat to vector control programmes [17].

This study focuses on finding the concentration of ivermectin that kills 50% of adult female *An. coluzzii* mosquitoes (LC_50_) that have imbibed ivermectin in a blood meal and the time to which median mortality is achieved at given ivermectin concentrations by measuring daily survival. It also aims to identify the concentration threshold where ivermectin will significantly impact fecundity and egg laying over 14 days in *An. coluzzii* exposed to concentrations of ivermectin in blood meals that represent a range of plausible venous blood concentrations in treated humans. Understanding the lethal and sublethal dose-response effect of ivermectin in this major malaria vector will provide further clarity on whether there is a significant difference in efficacy of the drug across different concentrations on both mortality and fecundity. Additionally, low doses of ivermectin administered orally in MDA may not provide significant mortality over time, whereas sublethal impacts on fecundity at low doses may be sufficient to both lower the vector population and limit spread of ivermectin resistance, with important implications for implementation of this approach in disease control.

## Methods

### Mosquito colony

An *An. coluzzii* laboratory strain was used and maintained at 27 ± 5 °C and 80 ± 10% relative humidity with a 12-h light:dark photoperiod. Adult mosquitoes were held in 30 × 30 × 30-cm cages and fed ad libitum via cotton soaked in 10% sucrose solution. Larvae were fed TetraMin™ fish flakes. All mosquitoes were blood fed with defibrinated horse blood heated to ~ 37 °C via a feeding system using a swine intestine membrane (Hemotek, UK), at 4–5 days post-emergence and during the 12 h light photoperiod.

### Drugs and reagents

An ivermectin formulation using commercially available powdered ivermectin (≥ 95%, MP Biomedicals™, Fisher Scientific) was prepared in dimethyl sulphoxide (DMSO) and phosphate-buffered saline (PBS). Ivermectin was first dissolved in DMSO at a concentration of 10 mg/ml and stored at 4 °C. The 10 mg/ml stock was then diluted in PBS to create working stock concentrations of 1 mg/ml and 0.001 mg/ml. Ivermectin concentration of the 0.001 mg/ml DMSO/PBS formulation was verified by high-performance liquid chromatography (HPLC) compared to a standard ivermectin reference solution. To achieve the desired concentration of ivermectin in a 5 ml blood-meal volume, the 0.001 mg/ml working stock solution was diluted in horse blood to the desired concentration for each ivermectin blood-meal treatment (Table 1). The control of 0 ng/ml stock solution contained PBS with DMSO at a concentration equivalent to the concentration of DMSO and PBS in the highest concentration DMSO/PBS ivermectin preparation (100 ng/ml), which was equivalent to 0.01% (v/v) DMSO in 1 × PBS.

**Table 1:** Preparation of ivermectin concentrations in blood meals using ivermectin DMSO/PBS formulation diluted in horse blood and experimental sample sizes

Ivermectin concentration in blood meal (ng/ml)	Ivermectin dilution in horse blood	No. of mosquitoes fed per replicate		
		1st replicate	2nd replicate	3rd replicate
0	500 μl from 0.01% (v/v) DMSO/PBS stock	26	25	25
10	50 μl from 0.001 mg/ml stock in 5 ml	22	25	25
15	75 μl from 0.001 mg/ml stock in 5 ml	22	25	25
25	125 μl from 0.001 mg/ml stock in 5 ml	25	25	25
50	250 μl from 0.001 mg/ml stock in 5 ml	24	25	25
75	375 μl from 0.001 mg/ml stock in 5 ml	25	25	25
100	500 μl from 0.001 mg/ml stock in 5 ml	23	25	25

### Blood feeding and ivermectin administration

Defibrinated horse blood (Sigma) was used for all blood-meal preparations. Prior to blood feeding, mosquitoes were sugar starved for 4 h. Female mosquitoes were isolated 5–6 days post-emergence in rearing cages. For each ivermectin concentration and the control, approximately 25 mosquitoes were exposed to a blood meal for 30 min. Three replicates of this were repeated for each treatment and control. Blood contained ivermectin at the following concentrations: 0 ng/ml (control), 10 ng/ml, 15 ng/ml, 25 ng/ml, 50 ng/ml, 75 ng/ml, and 100 ng/ml.

### Mosquito survival

After blood feeding, fully engorged females were removed from the cage and placed individually into empty observation vials (Sterilin 30-ml universal container, Thermo Scientific) and provided with cotton wool soaked in 10% sugar solution. Mortality was recorded every 24 h for 14 days post-blood feeding. This period was chosen to reflect the extrinsic incubation period of *Plasmodium falciparum*, which is typically 7–10 days [23], and because *Anopheles* mosquitoes can typically be expected to live up to 14 days in the wild [24]. Mosquitoes were regarded as dead if they were laying on the bottom of the vial, unable to move. Mosquitoes that were able to fly, stand but not fly, or had moving limbs were recorded as alive. Three replicates of approximately 25 mosquitoes per replicate were performed for each ivermectin treatment and the control.

### Egg production

Five days post-blood feed, any surviving mosquitoes were carefully removed from observation vials and transferred into individual oviposition vials (Sterilin 30-ml universal container, Thermo Scientific) using a mouth aspirator. Mosquitoes were provided with cotton wool soaked in 10% sugar solution and water-dampened cotton wool covered with a 2-cm diameter filter paper circle as an egg-laying substrate. Vials were checked daily for the presence of eggs. When eggs were identified, the filter paper was removed, and any eggs were counted using a stereoscopic dissection microscope at 2× magnification. Filter paper was then replaced to allow for repeat egg laying and the vial marked to identify the female as having oviposited. Both the number of ovipositing females and corresponding number of eggs laid were recorded for each mosquito, for each day, up to day 14.

### Statistical analysis

The LC_50_ of ivermectin was calculated using log-probit regression analysis [11] of daily mortality in SPSS version 28.0.1 (IBM SPSS). To assess the effect of ivermectin concentration on survival of adult females, Kaplan-Meier survival analysis and log-rank (Mantel-Cox) regression analysis were performed using survival analysis in GraphPad Prism version 8. Dose-response curves were generated using logit analysis in RStudio version 2023.03.386. To determine whether mean survival at each concentration was significantly different, generalised linear models (GLM) followed by pairwise comparisons using post hoc Tukey tests (95% confidence interval) in RStudio version 2023.03.386 and Minitab (version 18) were performed.

Egg production data were not normally distributed according to Shapiro-Wilk test; therefore, nonparametric Kruskal-Wallis analysis of variance (ANOVA) was used to examine variation in number of eggs laid and number of ovipositing females between ivermectin concentrations. A post hoc Dunnett’s multiple comparisons test was used to assess whether there was any significant difference in the number of eggs produced per surviving female between ivermectin treatments groups and the 0 ng/ml control group and the effect of ivermectin on the number of ovipositing females between ivermectin treatments groups and the 0 ng/ml control group. Egg production data were analysed in GraphPad Prism version 8.

## Results

### Mosquito mortality

**Fig. 1:**
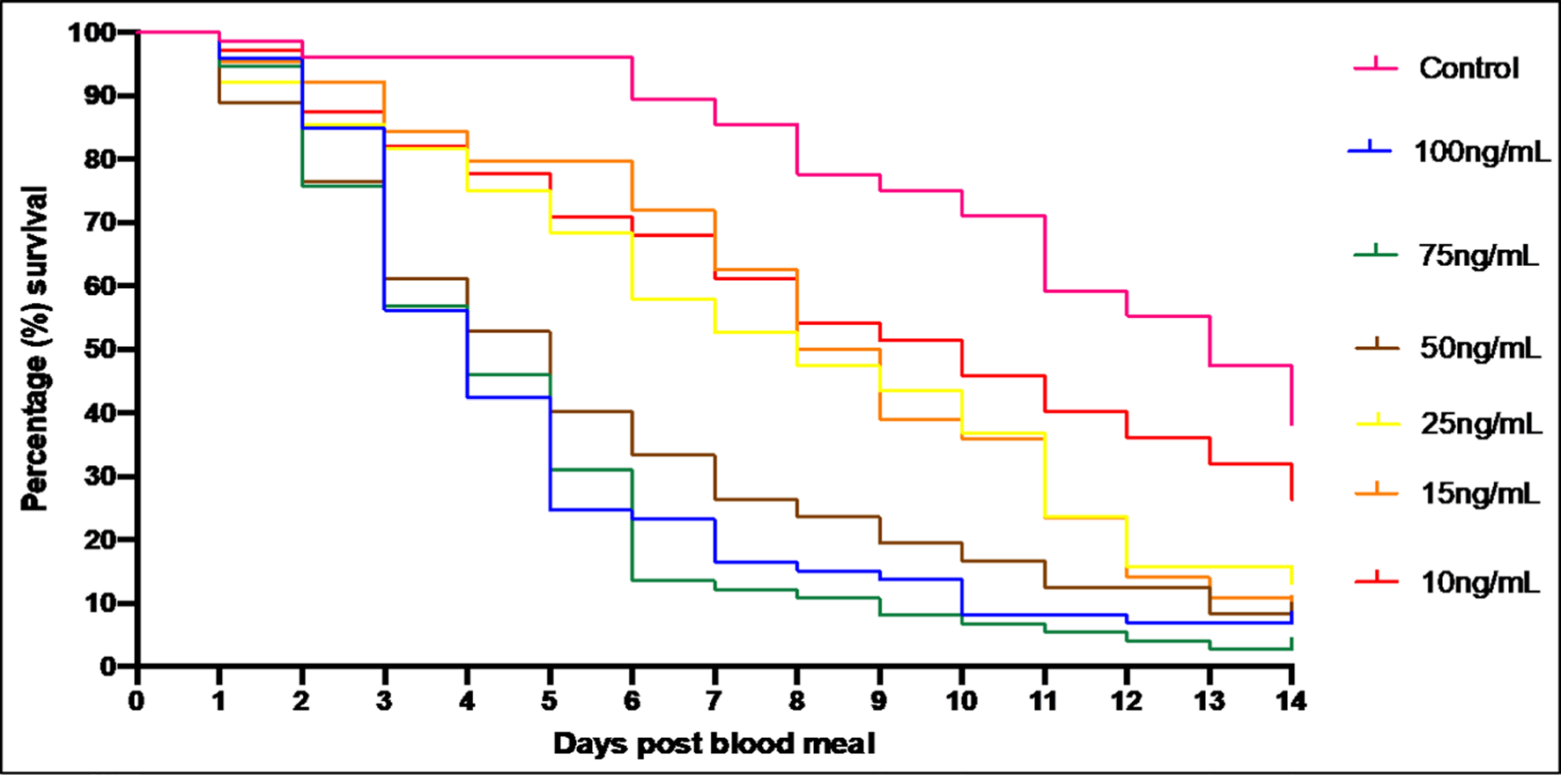
Fourteen-day Kaplan-Meier survival curves showing the daily survival of *Anopheles coluzzii* females after ingestion of ivermectin at various concentrations

In total, 450 *An. coluzzii* adult females took blood meals and were used to assess the oral toxicity of membrane-fed ivermectin by measuring mortality. Kaplan-Meier survival curves were produced for each ivermectin treatment (0–100 ng/ml) (Fig. 1). Table 2 shows the LC_50_ of *An. coluzzii* at day 3, 7, and 14 using logit regression analysis.

**Table 2:** Lethal concentrations (LC) with 95% confidence intervals (*df* = 4) of ivermectin for *Anopheles coluzzii* at 3, 7, and 14days post-blood feeding, calculated using log-probit analysis

Ivermectin concentration (ng/ml) (95% CI)			
LC (%)	Day 3	Day 7	Day 14
20	1.27 (0.20–3.16)	0.969 (0.34–1.87)	0.144 (0.00–0.93)
50	122.06 (82.57–248.88)	19.769 (15.61–23.94)	1.316 (0.029–3.91)
90	2108.27 (726.78–18,487.69)	129.795 (93.91–211.44)	38.396 (24.79–97.82)

**Fig. 2:**
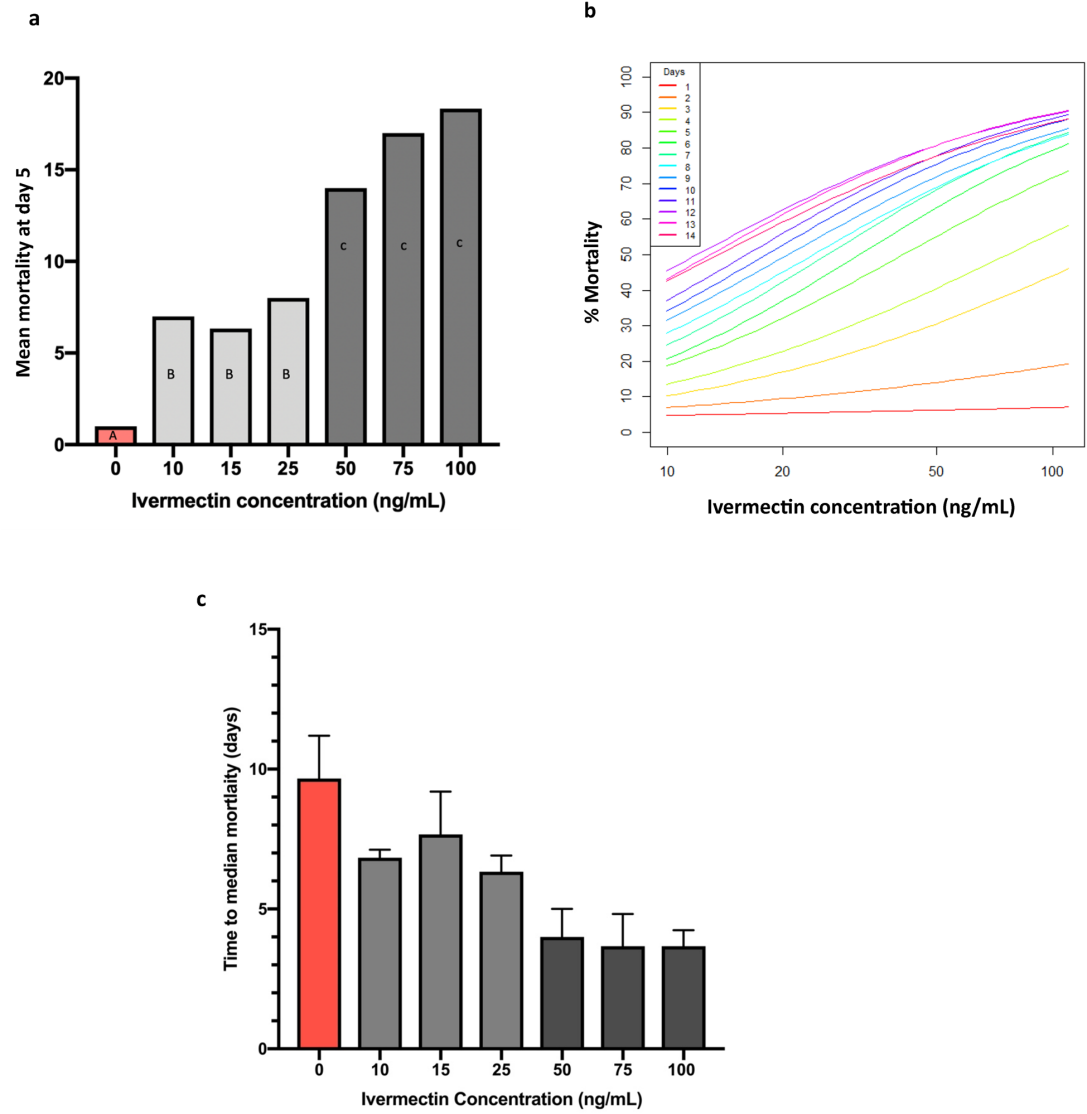
Post-ivermectin ingestion mortality. **a** Day 5 mean mortality; GLM with post hoc Tukey pairwise comparison and 95% confidence of mean mortality between ivermectin concentrations on day 5 post-blood meal. Bars with the same letter are not significantly different from one another (*p* < 0.05). **b** Logit dose-response curves for each ivermectin concentration by day, correcting for control mortality. **c** Time to median mortality in days at each given ivermectin concentration, from 0 ng/ml to 100 ng/ml, with error bars showing standard deviation across the three replicates performed

Ivermectin ingestion significantly reduced the survivorship of mosquitoes over 14 days at all concentrations tested (Mantel-Cox log-rank test, *df* = 6, *p* < 0.0001). Survival was high in the 0 ng/ml ivermectin control group until day 11, when the impact of age-related mortality could start to be seen. Survival was similar at all concentrations relative to the control until day 2–3 post-blood feed, where survival decreased notably in ivermectin-treated groups. At day 3, almost 50% of females in the 75 ng/ml and 100 ng/ml treatment groups had died. By day 5, substantial mortality occurred in a concentration-dependent manner, such that mortality responses could be defined by grouping into “low” (10 ng/ml, 15 ng/ml, 25 ng/ml) and “high” (50 ng/ml, 75 ng/ml, 100 ng/ml) ivermectin concentration treatment groups (Fig. 1). Specifically, mean mortality across all concentrations at day 5 was significantly different from the control (GLM, Tukey, *p* ≤ 0.05, Fig. 2a); however, no significant difference was found between incremental doses when comparing mean mortality within the low ivermectin concentration range or between incremental doses when comparing mean mortality within the high ivermectin concentration range (GLM, Tukey, *p* ≤ 0.05, Fig. 2a). This indicates that at 5 days post-ivermectin blood meal, there is a clear distinction in mean survival between the higher and lower ivermectin concentrations tested in this study compared to control but that low ivermectin concentrations (10 ng/ml, 15 ng/ml, and 25 ng/ml) share a similar potency in reduction on survival, and likewise high concentrations (50 ng/ml, 75 ng/ml, and 100 ng/ml) cause a similar reduction in survival of *An. coluzzii* adult females (Fig. 2a) that is not significantly different between doses.

From the pairwise comparison of mean mortality across 14 days, all concentrations were  significantly different from 0 ng/ml control (GLM, Tukey, *p* ≤ 0.05). The final % mortality at the end of the study was relatively similar for all treatments compared to control, except for 10 ng/ml (Fig. 1). The difference in overall mortality between doses 15 ng/ml–100 ng/ml is not huge. After 14 days, a 15 ng/ml dose was shown to kill around 85% of females compared to 0 ng/ml treatment, where 60% of females did not survive to day 14. Increasing the dose to 100 ng/ml increases this mortality to 95% at day 14, which for the difference in dose does not show a proportional increase. No significant difference was found between mean mortality of incremental doses except between 10 ng/ml and the higher ivermectin concentration doses, 50 ng/ml, 75 ng/ml, and 100 ng/ml (GLM, Tukey, *p* ≤ 0.05, Table 3,). The lethal concentrations of ivermectin in *An. coluzzii* was found to be dependent on time after ingestion, with a 79% lower LC_50_ at day 7 post-ingestion than at day 3. Time to median mortality (days) was calculated for each ivermectin concentration. In this study, the time to median morality for the control group was 9.6 days. Time to median mortality for 50 ng/ml, 75 ng/ml, and 100 ng/ml ivermectin concentrations was similar at 4, 3.6, and 3.6 days respectively (Fig. 2c). For 10 ng/ml, 15 ng/ml, and 25 ng/ml, the time to median mortality was closer to that of the control group but still distinctly less, at 6.8, 7.6, and 6.3 days respectively (Fig. 2c) and again similar between the lower concentration ivermectin doses. For all ivermectin concentrations, the time to median mortality was less than for the control group in all three replicates performed.

**Table 3:** GLM with post hoc Tukey pairwise comparison (95% confidence) of mean mortality between ivermectin concentrations over 14 days

Ivermectin concentration	Mean mortality	Grouping
0 ng/ml	19.6667	A
10 ng/ml	14.7333	B
15 ng/ml	13.4000	B, C
25 ng/ml	13.2444	B, C
50 ng/ml	9.7556	C, D
75 ng/ml	8.2667	D
100 ng/ml	7.8889	D

Means with the same letter are not significantly different at *p* > 0.05

### Sublethal impacts of ivermectin

Oviposition was low overall in all treatment groups, with the highest rates of oviposition recorded in the control group and 10 ng/ml, where 31.9% and 32.0% of surviving females, respectively, went on to oviposit (Table 4). Ivermectin ingestion was found to significantly decrease both the total number of ovipositing females (Kruskal-Wallis ANOVA, *p* < 0.0001) and number of eggs laid per individual (Kruskal-Wallis ANOVA, *p* < 0.0001). Fecundity, measured by number of ovipositing females and number of eggs laid per female, in the lower concentration groups (10–25 ng/ml) was found to be similar to that of the 0 ng/ml control group. However, at higher concentrations between 50 ng/ml and 100 ng/ml, both the number of ovipositing females and number of eggs laid per female were significantly reduced.

**Table 4:** Fecundity of *Anopheles coluzzii* following ingestion of ivermectin at various concentrations showing egg production and oviposition across the three-replicate experiment

Ivermectin (ng/ml)	No. of mosquitoes fed	% of surviving females on day 5	Mean no. of ovipositing females ± SEM	% oviposited of total fed	% oviposited of surviving females	Mean no. of eggs laid ± SEM
0	76	72 (94.7)	7.6 ± 2.9	30.2	31.9	489.7 ± 208.7
10	72	50 (69.4)	5.3 ± 1.4	22.2	32.0	381.0 ± 197.4
15	72	53 (73.0)	5.3 ± 2.4	22.2	30.1	320.7 ± 179.5
25	75	51 (68.0)	4.0 ± 1.0	16.0	23.5	247.7 ± 93.51
50	74	31 (41.0)	0.6 ± 0.6	2.7	6.4	8.0 ± 8.0
75	75	24 (32.0)	0 ± 0	0	0	0 ± 0
100	73	18 (24.7)	0 ± 0	0	0	0 ± 0

**Fig. 3:**
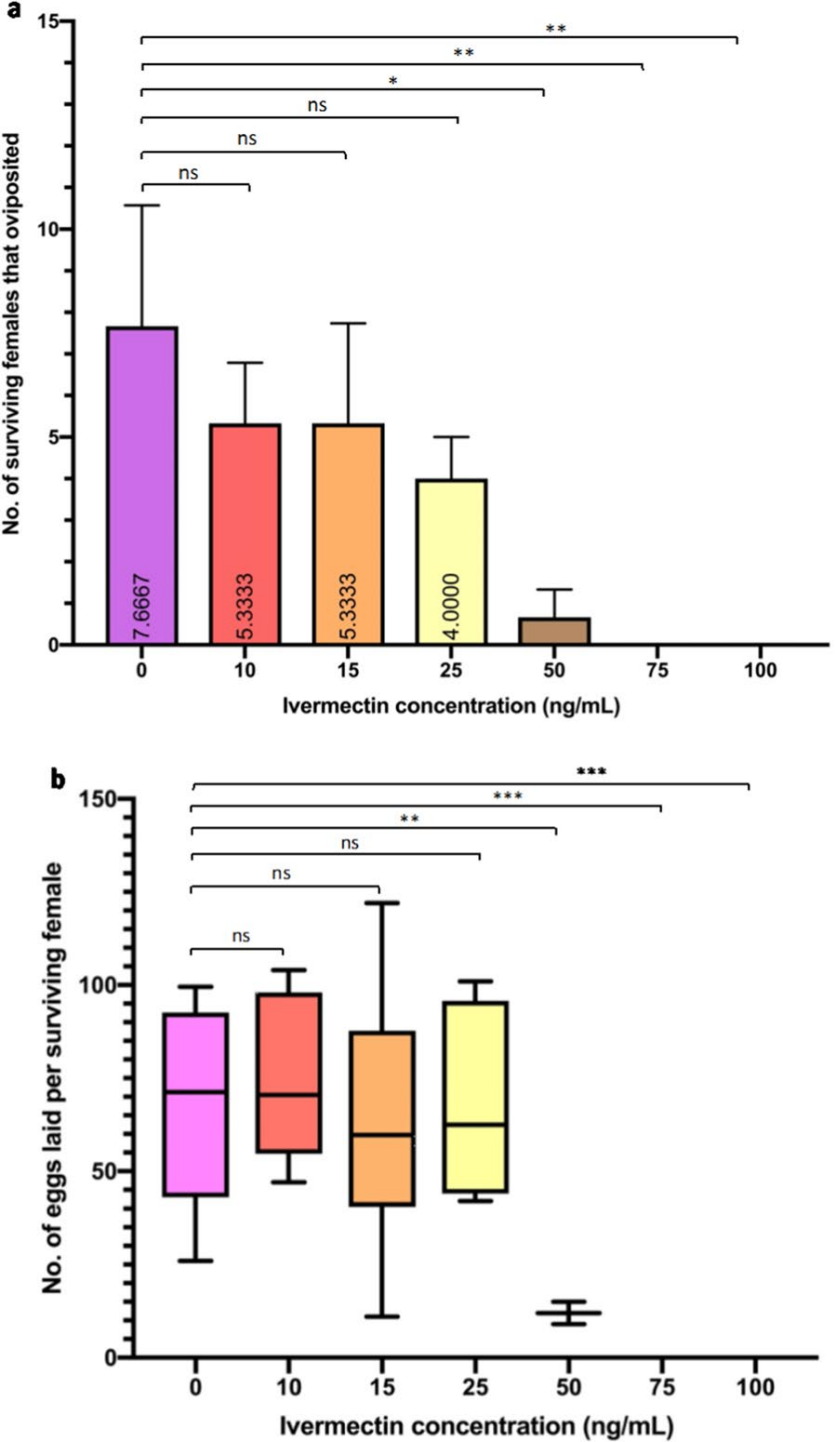
**a, b** Fecundity of *Anopheles coluzzii* following ivermectin ingestion from a blood meal. **a** Number of surviving females that went on to oviposit from day 5 to day 14. **b** Mean number of eggs laid per surviving female. Asterisks represent the level of statistical difference from the control group (0 ng/ml). *P < 0.05, **P < 0.01, ***P < 0.001, ****P < 0.0001

Any oviposition that was observed happened following day 5 post-blood feed. Control females oviposited between day 6 and day 14 post-blood feed. Females exposed to 25 ng/ml, 15 ng/ml, and 10 ng/ml ivermectin blood meal oviposited between day 5 and day 14; no oviposition was observed in surviving females from either the 100 ng/ml or 75 ng/ml ivermectin treatment groups. Of the three replicates performed, two females in the first replicate laid eggs from the 50 ng/ml treatment group on day 8 post-blood feed; no further egg laying was observed in the 50 ng/ml treatment group. When comparing the number of eggs laid per individual between ivermectin treatment groups to control, no significant difference was found for the number of eggs laid by females exposed to 25 ng/ml, 15 ng/ml, and 10 ng/ml ivermectin blood meals (Dunn’s multiple comparisons *p* = 0.3158, *p* > 0.9999, *p* = 0.8707 respectively). However a significant difference was found for females exposed to 50 ng/ml, 75 ng/ml, and 100 ng/ml ivermectin (Dunn’s multiple comparisons, **p = 0.0021, ***p = 0.0002, ***p = 0.002 respectively, Fig. 3). Similarly, no significant difference was found for the number of ovipositing females exposed to 25 ng/ml, 15 ng/ml, and 10 ng/ml ivermectin blood meals compared to control, but a significant difference (Dunn’s multiple comparisons *p = 0.0448, **p = 0.0100, **p = 0.0100 respectively, Fig. 3) was found for the number of ovipositing females exposed to 50 ng/ml, 75 ng/ml, and 100 ng/ml ivermectin compared to control.

## Discussion

Results from this study demonstrate that ivermectin concentrations as low as 10 ng/ml have a significant effect on mortality of *An. coluzzii* and reduce survival over 14 days post-blood feeding. They also show clear evidence of ivermectin’s sub-lethal effects on *An. coluzzii*, which could influence vector population dynamics. The lethal dose (LC_50_) of ivermectin was 19.7 ng/ml, higher than previously reported in an in vitro study of *An. gambiae*, which calculated the 7-day LC_50_ to be 15.9 ng/ml [16] from membrane-fed ivermectin in blood. In comparison, the 7-day LC_50_ reported across other *Anopheles* species ranges from 3.35 ng/ml to 55.6 ng/ml [12, 16, 25–27]. The time to median mortality for ivermectin concentrations ≥ 50 ng/ml was ≤ 4 days compared to 9.6 days for 0 n/ml control and 6.3–7.6 days for ivermectin concentrations of ≤ 25 ng/ml (Fig. 2c); higher ivermectin concentrations in the range 50 ng/ml–100 ng/ml therefore were found to induce a time to median mortality that was 61% faster than that of control. Conversely, lower ivermectin concentrations between 10 ng/ml and 25 ng/ml were less efficacious than higher concentrations; however, they did also reduce time to median mortality by 28% when compared against time to naturally occurring death in untreated mosquitoes.

Mass drug administration campaigns using ivermectin for onchocerciasis and lymphatic filariasis typically administer a single oral dose at approximately 150–200 ug/kg. Ivermectin at these oral doses has been shown to have an insecticidal effect on *Anopheles* populations feeding on treated patients in vivo [28]; however, the toxicity is short lived, at 7 days or less. The efficacy of ivermectin is related to pharmacokinetics associated with certain dosing regimens and has been linked to the C_max_ recorded following these doses. C_max_ is a pharmacokinetic measurement that describes the highest plasma concentration of a drug measured following administration of a single dose. The highest C_max_ recorded for ivermectin in venous blood is reported as 105.2 ng/ml and was achieved using a high oral dose of 300 μg/kg and 600 μg/kg a day given over 3 days [14]. From a single 12-mg dose representing a mean 165 μg/kg weight-adjusted dose, given in 3-mg tablets, the mean peak plasma concentration of ivermectin recorded in fasting healthy male volunteers was 46.5 ng/ml [29]. Typical oral dosing for ivermectin ranges between 150 μg/kg and 200 μg/kg. The 7-day LC_50_ found in this study, 19.7 ng/ml, is therefore well within the highest reported venous C_max_ recorded after high oral dose, and the lowest plasma C_max_ achieved from a relatively standard mean 165 μg/kg oral dose, indicating the feasibility of ivermectin as a mosquitocidal in *An. coluzzii* when dosed clinically. Given the range of LC_50_ reported across the *Anopheles* species (3.35 ng/ml to 55.6 ng/ml), and the LC_50_ estimated from this study, which falls in the lower half of this range, to achieve universal lethal impact across all *Anopheles* species that have been tested, the concentration at which ivermectin should be maintained in a blood meal would need to be ≥ 55.6 ng/ml. Time to C_max_ (T_max_) for ivermectin is approximately 4 h, and the plasma half-life of ivermectin administered orally is approximately 18 h [30]. Considering the oral plasma half-life of ivermectin, and the C_max_ of a 165 μg/kg oral dose, maintaining an ivermectin concentration of 55.6 ng/ml for sufficient time to be effective in reducing vector populations, and therefore impact malaria transmission, is unlikely with current formulations available.

The mode of transfer of ivermectin to mosquitoes is, however, important to consider when drawing comparisons of in vivo and in vitro ivermectin dosing because of differences in the concentration of ivermectin in a blood meal via membrane feeding versus skin feeding. The mortality effect of ivermectin in *Anopheles* species has been reported for doses ranging between 150 and 300 μg/kg, which have been measured by venous blood concentration, resulting in lower predicted LC_50_ values compared to in vitro membrane-feeding experiments using ivermectin concentrations prepared in blood meals. The reason for this difference is likely due to the pharmacokinetics of ivermectin and its physicochemical properties. As a lipophilic compound, ivermectin accumulates in dermal and adipose fatty tissues and will therefore reach higher concentrations in skin capillaries than in venous plasma [31] from which C_max_ is calculated. As mosquitoes imbibe blood from subdermal capillaries, they are likely to ingest higher concentrations of ivermectin when skin feeding than those that would be found if quantified from venous plasma [30]. This means that the effects of ivermectin on mosquito survival based on concentrations derived from venous blood in in vivo studies may not be directly comparable to the impacts on survival achieved with membrane-fed ivermectin concentrations in vitro, although a recent study found similar mosquitocidal effects of ivermectin when comparing membrane feeding to direct skin feeding [25]. In addition, the lethal effects of ivermectin metabolites have more recently been tested in *Anopheles stephensi* and found to be potentially responsible for the “post-ivermectin’ effect seen in mosquitoes that have been skin-fed from ivermectin-treated patients [32].

Alongside its lethal effects, ivermectin has the additional capacity to cause sublethal effects in mosquitoes. These include effects on fecundity, which in the context of vector control could provide additional benefits in reducing the size of mosquito populations through inhibition of egg laying in surviving females. In this study, higher concentrations (50–100 ng/ml) impacted fecundity by reducing the number of females that oviposited and decreasing the number of eggs laid by those that did. In fact, none of the surviving females exposed to 75 ng/ml or 100 ng/ml went on to lay any eggs at all, despite taking a full blood meal; lower doses (10–25 ng/ml) did not significantly reduce either of these measures of fecundity. Although ivermectin has a different mode of action to insecticides currently used in public health, meaning it is likely to be effective against insecticide-resistant populations, a potential metabolic mechanism for resistance to ivermectin has been identified in *An. gambiae* s.s. [33]. Resistance to ivermectin is a potential consequence of exposing mosquito vectors through MDA to levels of ivermectin that are not maintained at mosquitocidal levels. It is therefore important to deduce concentrations that are sufficient to decrease fecundity as both a measure of residual ivermectin effect and limit the potential for ivermectin resistance in subsequent mosquito generations. The C_max_ of 46.5 ng/ml achieved from a standard 12 mg/165 μg/kg dose is unlikely to significantly reduce fecundity in *An. coluzzii* and could potentially drive emergence of ivermectin resistance in progeny of surviving females.

Overall, it was shown that in the range of 10 ng/ml to 100 ng/ml, ivermectin has potential as a tool for curbing residual malaria transmission by significantly reducing survival of the malaria vector *An. coluzzii* within the extrinsic incubation period of *P. falciparum*. Concentrations of ≥ 50 ng/ml, which are within the C_max_ achieved by higher oral doses of ≥ 600 μg/kg, caused significant sublethal impacts on mosquito reproduction. However, these were not observed at lower concentrations equivalent to what may be expected in patients treated with a standard 150–200 μg/kg oral dose, and so the prospect of resistance in target vectors should be considered. Current or recent field trials, such as BOHEMIA [9], however, report using higher or repeated doses of ivermectin for MDA, which may help mitigate this. Given the poor bioavailability of ivermectin via the oral route, changing the mode of administration and/or drug reformulation could see sustained release of ivermectin at concentrations where both lethal and sublethal impacts could be achieved across the *Anopheles* genus. Understanding the important difference in lethal and sublethal impacts between ivermectin concentrations will help to set goals for ivermectin release from formulation via alternative routes of administration that achieve both lethal and sublethal impacts with standard dosing. Other sublethal impacts in the form of behavioural changes, such as reduced host-seeking, and generational changes in fecundity, namely reduced egg-hatch rate from egg-laying survivors, may be additional mechanisms by which vector populations can be reduced and should be studied further in *An. coluzzii* and other malaria vector species.

## Conclusions

The present study demonstrated that ivermectin in the range of 10 ng/ml to 100 ng/ml significantly reduces survival in *An. coluzzii* over 14 days compared to control. The ability to reduce survival in *An. coluzzii* using membrane-fed ivermectin was found to be both concentration and time dependent, where the effect on survival can be broadly split into ‘higher’ and ‘lower’ ivermectin concentration groups, producing higher and lower percentage mortality over time in a concentration-dependent manner. This difference was seen clearly at 5 days post ivermectin ingestion at any given dose. At ≥ 50 ng/ml, sublethal impacts inhibit fecundity by preventing egg production in surviving females. The time to median mortality for ivermectin concentrations ≥ 50 ng/ml was ≤ 4 days compared to 9.6 days for 0 n/ml control and 6.3–7.6 days for ivermectin concentrations tested of ≤ 25 ng/ml. Adult *An. coluzzii* were found to oviposit from day 5 post-blood feed. For vector control purposes, concentrations should be maintained above 50 ng/ml to achieve sublethal impacts in *An. coluzzii* populations and at no less than 19.7 ng/ml to reduce a mosquito population by 50% in 7 days, above normal naturally occurring mortality and within the extrinsic incubation period of *P. falciparum*. However, the range of ivermectin concentrations showing lethal impacts across the *Anopheles* genus is wide. This indicates that the concentration of ivermectin is an important factor in achieving effective lethal and sublethal impacts in all species of malaria vectors and should be considered when developing ivermectin as a vector control tool. *Anopheles coluzzii* is one of the primary malaria vectors in sub-Saharan Africa and has more recently spread into main cities of Central Africa, which threatens current vector control programmes [17]. Current dosing regimens using ivermectin in oral doses designed for treating parasitic infections may not be appropriate for vector control in the long term as the duration of mosquitocidal concentrations in patient blood from these doses is short lived. The longer ivermectin remains in the blood at mosquito-killing concentrations, the more mosquitoes will be killed or disabled, effectively contributing to reducing malaria transmission [30]. Repurposing ivermectin as a mosquitocidal intervention may therefore require reformulation of the drug to improve bioavailability and achieve sustained mosquitocidal concentrations for all *Anopheles* malaria vectors while avoiding 3-day high-oral dose ivermectin regimens.

## Data Availability

The authors confirm that the data supporting the findings of this study are available within the article.

## References

[1] Khaligh FG, Jafari A, Silivanova E, Levchenko M, Rahimi B, Gholizadeh S (2021). Endectocides as a complementary intervention in the malaria control program: a systematic review. Syst Rev.

[2] Chaccour C, Lines J, Whitty CJM (2010). Effect of ivermectin on *Anopheles gambiae* mosquitoes fed on humans: the potential of oral insecticides in malaria control. J Infect Dis.

[3] Batiha GES, Alqahtani A, Ilesanmi OB, Saati AA, El-Mleeh A, Hetta HF (2020). Avermectin derivatives, pharmacokinetics, therapeutic and toxic dosages, mechanism of action, and their biological effects. Pharmaceuticals.

[4] Chaccour CJ, Kobylinski KC, Bassat Q, Bousema T, Drakeley C, Alonso P, Foy BD (2013). Ivermectin to redcuce malaria transmission:a research agenda for a promising new tool for elimination. Malar J.

[5] Laing R, Gillan V, Devaney E (2017). Ivermectin—old drug, new tricks?. Trends Parasitol.

[6] Atif M, Estrada-Mondragon A, Nguyen B, Lynch JW, Keramidas A (2017). Effects of glutamate and ivermectin on single glutamate-gated chloride channels of the parasitic nematode *H*. *contortus*. PLoS Pathog.

[7] Wolstenholme AJ (2012). Glutamate-gated chloride channels. J Biol Chem.

[8] WHO (2015). Global technical strategy for malaria 2016–2030.

[9] Chaccour C, Casellas A, Hammann F, Ruiz-Castillo P, Nicolas P, Montaña J (2023). BOHEMIA: broad one health endectocide-based malaria intervention in Africa—a phase III cluster-randomized, open-label, clinical trial to study the safety and efficacy of ivermectin mass drug administration to reduce malaria transmission in two African set. Trials.

[10] Kobylinski KC, Escobedo-Vargas KS, López-Sifuentes VM, Durand S, Smith ES, Baldeviano GC (2017). Ivermectin susceptibility, sporontocidal effect, and inhibition of time to re-feed in the Amazonian malaria vector Anopheles darlingi. Malar J.

[11] Hadlett M, Nagi SC, Sarkar M, Paine MJI, Weetman D (2021). High concentrations of membrane-fed ivermectin are required for substantial lethal and sublethal impacts on *Aedes*
*aegypti*. Parasit Vectors.

[12] Eba K, Habtewold T, Asefa L, Degefa T, Yewhalaw D, Duchateau L (2023). Effect of ivermectin^®^ on survivorship and fertility of *Anopheles*
*arabiensis* in Ethiopia: an in vitro study. Malar J.

[13] Mekuriaw W, Balkew M, Messenger LA, Yewhalaw D, Woyessa A, Massebo F (2019). The effect of ivermectin^®^ on fertility, fecundity and mortality of *Anopheles*
*arabiensis* fed on treated men in Ethiopia. Malar J.

[14] Smit MR, Ochomo EO, Aljayyoussi G, Kwambai TK, Abong’o BO, Chen T (2018). Safety and mosquitocidal efficacy of high-dose ivermectin when co-administered with dihydroartemisinin-piperaquine in Kenyan adults with uncomplicated malaria (IVERMAL): a randomised, double-blind, placebo-controlled trial. Lancet Infect Dis.

[15] Pinilla YT, CP Lopes S, Sampaio V, Andrade FS, Melo GC, Orfanó AS (2018). Promising approach to reducing Malaria transmission by ivermectin: sporontocidal effect against Plasmodium vivax in the South American vectors
* Anopheles
*
* aquasalis
* and Anopheles darlingi. PLoS Negl Trop Dis.

[16] Kobylinski KC, Foy BD, Richardson JH (2012). Ivermectin inhibits the sporogony of *Plasmodium*
*falciparum* in *Anopheles*
*gambiae*. Malar J.

[17] Vargas-Chavez C, Pendy NML, Nsango SE, Aguilera L, Ayala D, González J (2022). Transposable element variants and their potential adaptive impact in urban populations of the malaria vector *Anopheles*
*coluzzii*. Genome Res.

[18] Coetzee M, Hunt RH, Wilkerson R, Della Torre A, Coulibaly MB, Besansky NJ (2013). Anopheles coluzzii and Anopheles amharicus, new members of the
* Anopheles
*
* gambiae
* complex. Zootaxa.

[19] Perugini E, Guelbeogo WM, Calzetta M, Manzi S, Virgillito C, Caputo B (2020). Behavioural plasticity of *Anopheles*
*coluzzii* and *Anopheles*
*arabiensis* undermines LLIN community protective effect in a Sudanese-savannah village in Burkina Faso. Parasit Vectors.

[20] Wiebe A, Longbottom J, Gleave K, Shearer FM, Sinka ME, Massey NC (2017). Geographical distributions of African malaria vector sibling species and evidence for insecticide resistance. Malar J.

[21] Fouet C, Atkinson P, Kamdem C (2018). Human interventions: driving forces of mosquito evolution. Trends Parasitol.

[22] Vontas J, Grigoraki L, Morgan J, Tsakireli D, Fuseini G, Segura L (2018). Rapid selection of a pyrethroid metabolic enzyme CYP9K1 by operational malaria control activities. Proc Natl Acad Sci USA.

[23] Phillips MA, Burrows JN, Manyando C, Van Huijsduijnen RH, Van Voorhis WC, Wells TNC (2017). Malaria. Nat Rev Dis Primers.

[24] Adugna T, Getu E, Yewhelew D (2022). Parous rate and longevity of anophelines mosquitoes in bure district, northwestern Ethiopia. PLoS ONE.

[25] Smit MR, Ochomo EO, Aljayyoussi G, Kwambai TK, Abong’o BO, Bousema T (2019). Human direct skin feeding versus membrane feeding to assess the mosquitocidal efficacy of high-dose ivermectin (IVERMAL Trial). Clin Infect Dis.

[26] Ouédraogo AL, Bastiaens GJH, Tiono AB, Guelbéogo WM, Kobylinski KC, Ouédraogo A (2015). Efficacy and safety of the mosquitocidal drug ivermectin to prevent malaria transmission after treatment: a double-blind, randomized, clinical trial. Clin Infect Dis.

[27] Kobylinski KC, Deus KM, Butters MP, Hongyu T, Gray M, da Silva IM (2010). The effect of oral anthelmintics on the survivorship and re-feeding frequency of anthropophilic mosquito disease vectors. Acta Trop.

[28] Slater HC, Foy BD, Kobylinski K, Chaccour C, Watson OJ, Hellewell J (2020). Ivermectin as a novel complementary malaria control tool to reduce incidence and prevalence: a modelling study. Lancet Infect Dis.

[29] Merk Sharp & Dohme (Australia) Pty Ltd. Product information stromectol tablets (ivermectin 3 mg ) Stromectol^®^*Streptomyces**avermitilis* pharmacokinetics : onchocerciasis in adults. 2013.

[30] Chaccour C, Hammann F, Rabinovich NR, The World Health Organization (WHO) (2017). Ivermectin to reduce malaria transmission I. Pharmacokinetic and pharmacodynamic considerations regarding efficacy and safety. Malar J.

[31] Chaccour C, Barrio ÁI, Royo AGG, Urbistondo DM, Slater H, Hammann F (2015). Screening for an ivermectin slow-release formulation suitable for malaria vector control. Malar J.

[32] Kern C, Müller P, Chaccour C, Liechti ME, Hammann F, Duthaler U (2023). Pharmacokinetics of ivermectin metabolites and their activity against *Anopheles*
*stephensi* mosquitoes. Malar J.

[33] Nicolas P, Kiuru C, Wagah MG, Muturi M, Duthaler U, Hammann F (2021). Potential metabolic resistance mechanisms to ivermectin in *Anopheles*
*gambiae*: a synergist bioassay study. Parasit Vectors.

